# Detection of distinct glycosylation patterns on human γ-glutamyl transpeptidase 1 using antibody-lectin sandwich array (ALSA) technology

**DOI:** 10.1186/s12896-014-0101-0

**Published:** 2014-12-06

**Authors:** Matthew B West, Katie Partyka, Christa L Feasley, Kevin A Maupin, Indiwari Goppallawa, Christopher M West, Brian B Haab, Marie H Hanigan

**Affiliations:** Department of Cell Biology, University of Oklahoma Health Sciences Center, Oklahoma City, OK 73104 USA; Van Andel Research Institute, Grand Rapids, MI 49503 USA; Department of Biochemistry and Molecular Biology, University of Oklahoma Health Sciences Center, Oklahoma City, OK 73104 USA; Oklahoma Center for Medical Glycobiology, University of Oklahoma Health Sciences Center, Oklahoma City, OK 73104 USA; Stanton L. Young Biomedical Research Center, Rm. 264, 975 NE 10th St, Oklahoma City, OK 73104 USA

**Keywords:** γ-Glutamyl transpeptidase, Antibody-lectin sandwich arrays, *N*-glycans

## Abstract

**Background:**

γ-Glutamyl transpeptidase 1 (GGT1) is an *N*-glycosylated membrane protein that catabolizes extracellular glutathione and other γ-glutamyl-containing substrates. In a variety of disease states, including tumor formation, the enzyme is shed from the surface of the cell and can be detected in serum. The structures of the *N*-glycans on human GGT1 (hGGT1) have been shown to be tissue-specific. Tumor-specific changes in the glycans have also been observed, suggesting that the *N*-glycans on hGGT1 would be an important biomarker for detecting tumors and monitoring their progression during treatment. However, the large quantities of purified protein required to fully characterize the carbohydrate content poses a significant challenge for biomarker development. Herein, we investigated a new antibody-lectin sandwich array (ALSA) platform to determine whether this microanalytical technique could be applied to the characterization of *N*-glycan content of hGGT1 in complex biological samples.

**Results:**

Our data show that hGGT1 can be isolated from detergent extracted membrane proteins by binding to the ALSA platform. Probing hGGT1 with lectins enables characterization of the *N*-glycans. We probed hGGT1 from normal human liver tissue, normal human kidney tissue, and hGGT1 expressed in the yeast *Pichia pastoris*. The lectin binding patterns obtained with the ALSA platform are consistent with the hGGT1 *N*-glycan composition obtained from previous large-scale hGGT1 *N*-glycan characterizations from these sources. We also validate the implementation of the *Microcystis aeruginosa* lectin, microvirin, in this platform and provide refined evidence for its efficacy in specifically recognizing high-mannose-type *N*-glycans, a class of carbohydrate modification that is distinctive of hGGT1 expressed by many tumors.

**Conclusion:**

Using this microanalytical approach, we provide proof-of-concept for the implementation of ALSA in conducting high-throughput studies aimed at investigating disease-related changes in the glycosylation patterns on hGGT1 with the goal of enhancing clinical diagnoses and targeted treatment regimens.

**Electronic supplementary material:**

The online version of this article (doi:10.1186/s12896-014-0101-0) contains supplementary material, which is available to authorized users.

## Background

γ-Glutamyl Transpeptidase 1 (GGT1), is a clinically important enzyme that has been studied for over 95 years. Expressed on the surfaces of a variety of ductal and glandular epithelial cells throughout the body, GGT1 is strategically localized to catalyze the cleavage of glutathione in circulating fluids, allowing for the recovery of its constituent amino acids [1,2]. As such, GGT1 plays a key role in cysteine homeostasis [3]. However, abnormal expression and localization patterns of GGT1 are common in several human malignancies, including liver, kidney, prostate, pancreatic, ovarian, colon, and lung cancers [4-7]. The induction of GGT1 expression has been shown to protect tumors against oxidative stress by initiating the recovery of amino acids from extracellular glutathione for its intracellular synthesis, conferring GGT1-positive tumors with enhanced resistance to alkylating agents and other classes of chemotherapeutic drugs [8-10]. While elevated serum levels of GGT1 associated with liver and pancreatic cancers have been used as a diagnostic marker for monitoring disease progression and response to clinical intervention, its utility as a pre-symptomatic cancer biomarker has been hampered by the fact that high serum GGT1 levels are also observed in non-malignant diseases, ranging from alcoholic hepatitis to myocardial infarction [11-13]. Thus, the potential of serum GGT1 as a diagnostic marker of tumor formation and progression has been discounted historically.

Human GGT1 (hGGT1, Swiss-Prot: P19440), is a type II membrane-bound glycoprotein composed of two asymmetric subunits, possessing seven potential *N*-glycosylation sites [sequon N*X*(S/T), *X* ≠ P], with six sites on the large subunit and a single site on the small subunit [14-16]. Using high-resolution mass spectrometry, we have previously shown that all seven of these asparagine residues are capable of being glycosylated when hGGT1 is expressed in normal human tissues and have demonstrated that the structure and composition of the *N*-glycans vary in a tissue-specific manner [17]. While it has long been appreciated that the structural compositions of the *N*-glycans on hGGT1 change during malignant transformation, the large quantities of the tumor-derived glycoprotein required to characterize its carbohydrate content has posed a significant challenge for differential analyses [18-25]. Moreover, conventional methods, such as comparative lectin blotting or affinity chromatography coupled with mass spectrometry are labor-intensive and sample-exhaustive. Thus, the development of a more facile, high-throughput analytical method for reproducibly detecting differences in *N*-glycan content on small quantities of hGGT1 in complex biological samples would be of considerable benefit for investigative and diagnostic studies.

A promising emerging methodology for potentially addressing each of these intrinsic obstacles is antibody-lectin sandwich array (ALSA) technology [26]. Adapting existing multiplexed protein technology, ALSA couples the immuno-capture of glycoproteins from biological samples, using target-specific antibodies, with the ability to interrogate the bound target with a variety of biotinylated probes. Thus, a diverse group of lectins or glycan binding antibodies can be used to simultaneously probe for a variety of carbohydrate moieties or glycoepitopes on a glycoprotein of interest with a very small amount of starting material. Unlike most large-scale approaches, this methodology introduces an objective quantitative component that enhances the interpretative value associated with the qualitative changes that it detects. The amount of fluorophore-labeled streptavidin bound by a specific biotinylated probe at each capture site is directly related to the prevalence of a particular glycan modification [27]. Therefore, considerable compositional information can be gleaned from a single, small-scale sample preparation, a feature of ALSA which has been widely exploited for a diverse array of pathologies [28-30].

In recent years, glycans have experienced a renaissance as potential biomarkers of cancer and other human pathologies [31-34]. A great deal of effort has been devoted to developing techniques for characterizing and cataloging changes in disease-related glycosylation patterns. Historically, however, methodological approaches that have exploited the use of glycan binding proteins [35] have required relatively large samples for conducting discriminatory comparisons, and as a result, primary screening using glycan binding proteins could exhaust all of a sample before the optimal discriminatory lectin is even identified [36]. While the use of alternative complementary approaches, such as mass spectrometry, can provide finer structural resolution of glycosylation changes within individual samples, these techniques are not optimal for making simultaneous quantitative comparisons of glycan content between multiple sample pools, thus limiting their utility for primary biomarker screening [37]. The recent advent of ALSA has attempted to address these inherent technical limitations.

In the current study, we investigated whether the microanalytical advantages of the ALSA platform could be applied to the differential characterization of hGGT1 purified from disparate sources and bearing distinct, pre-defined *N*-glycan content. We show that immuno-captured hGGT1, originating from microgram quantities of either normal human liver tissue, normal human kidney tissue, or from hGGT1 expressed in the yeast *Pichia pastoris*, exhibits distinct ALSA binding patterns that complement results obtained from previous large-scale hGGT1 *N*-glycan characterizations from these sources. We also demonstrate the first use of the *Microcystis aeruginosa* lectin, microvirin (MVN), in this type of screening assay and provide evidence for its utility in specifically recognizing high-mannose-type *N*-glycans on hGGT1, a modification which is apparently unique to hGGT1 isoforms expressed in malignant kidney and liver tissues [19,21]. Hence, we provide proof of principle support for the implementation of ALSA in carrying out future high-throughput studies aimed at investigating changes in the glycosylation patterns on hGGT1 associated with human pathophysiological processes. We anticipate that this approach can be applied to developing primary diagnostic screening strategies and/or the development of a hGGT1 biomarker in studies evaluating therapeutic intervention.

## Results

### Glycan microarray analysis to determine the fine carbohydrate binding specificity of microvirin

**Figure 1:**
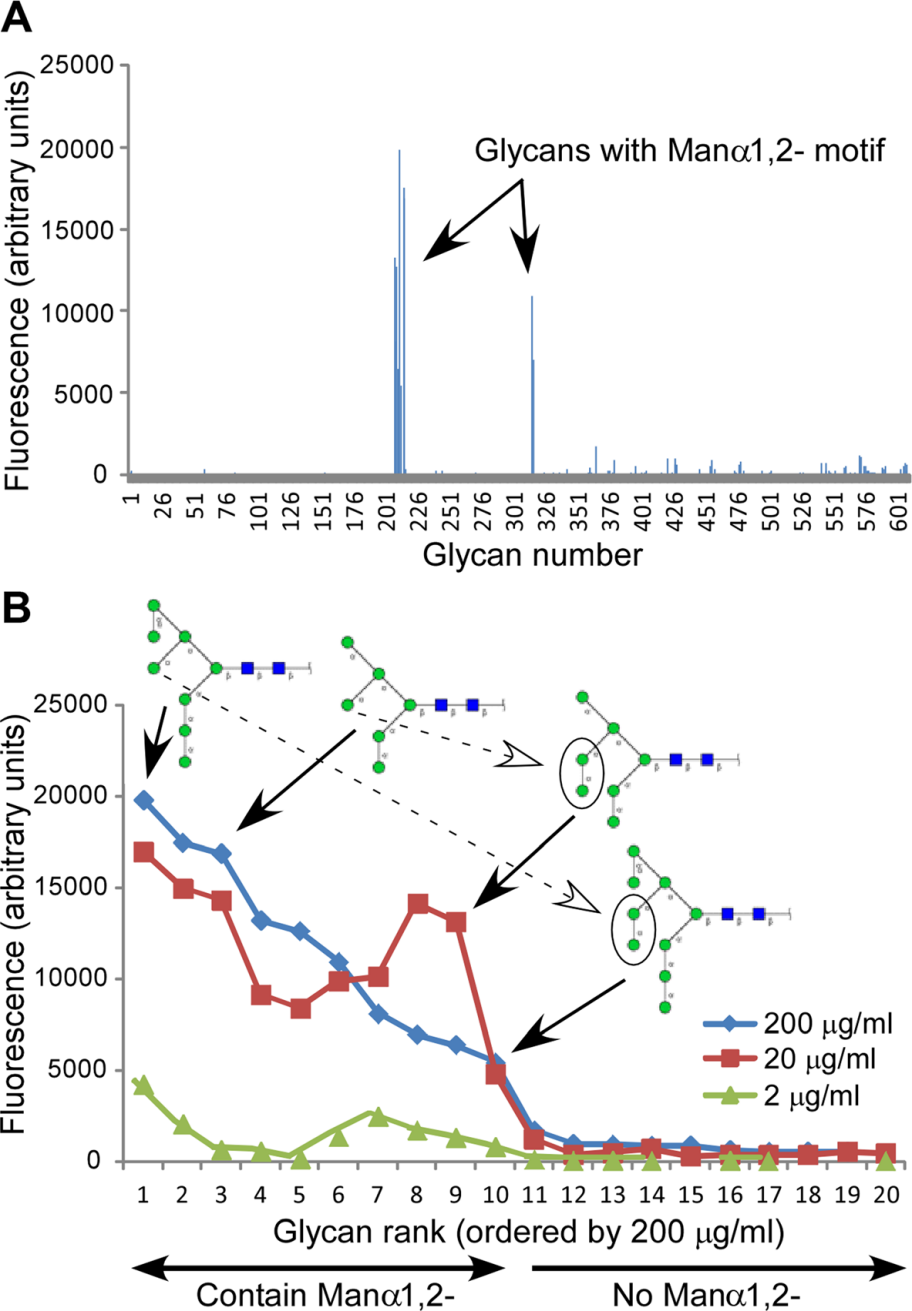
**Glycan array analysis of MVN CRD specificity. (A)** Binding profile of MVN at 200 μg/mL, using the CFG array V5.0 harboring 611 different carbohydrate structures. Relative fluorescence units reflect relative affinities toward the corresponding glycan. Glycans bound by MVN are indicated by their CFG array numbers (see Table 1). The diagram is based on the primary CFG data spreadsheets, which can be accessed at http://www.functionalglycomics.org/glycomics/publicdata/selectedScreens.jsp (Glycan Array #2340). **(B)** MVN glycan array binding at 2, 20, 200 μg/mL as ranked by fluorescence intensity (from 200 μg/mL data set) and apparent binding affinity as determined by Outlier Motif Analysis. Binding was considered positive if the RFU value was greater than the primary fluorescence threshold, calculated to be 2,319 RFU (see Figure 2). All 11 carbohydrates with above-threshold RFU values contained a Manα(1-2)Man- determinant, and the four shown illustrate the binding affinity differences that could be attributed to presence of the central arm Manα(1-2)Man-disaccharide. Symbols: *blue box*, GlcNAc; *green circle*, mannose.

**Figure 2:**
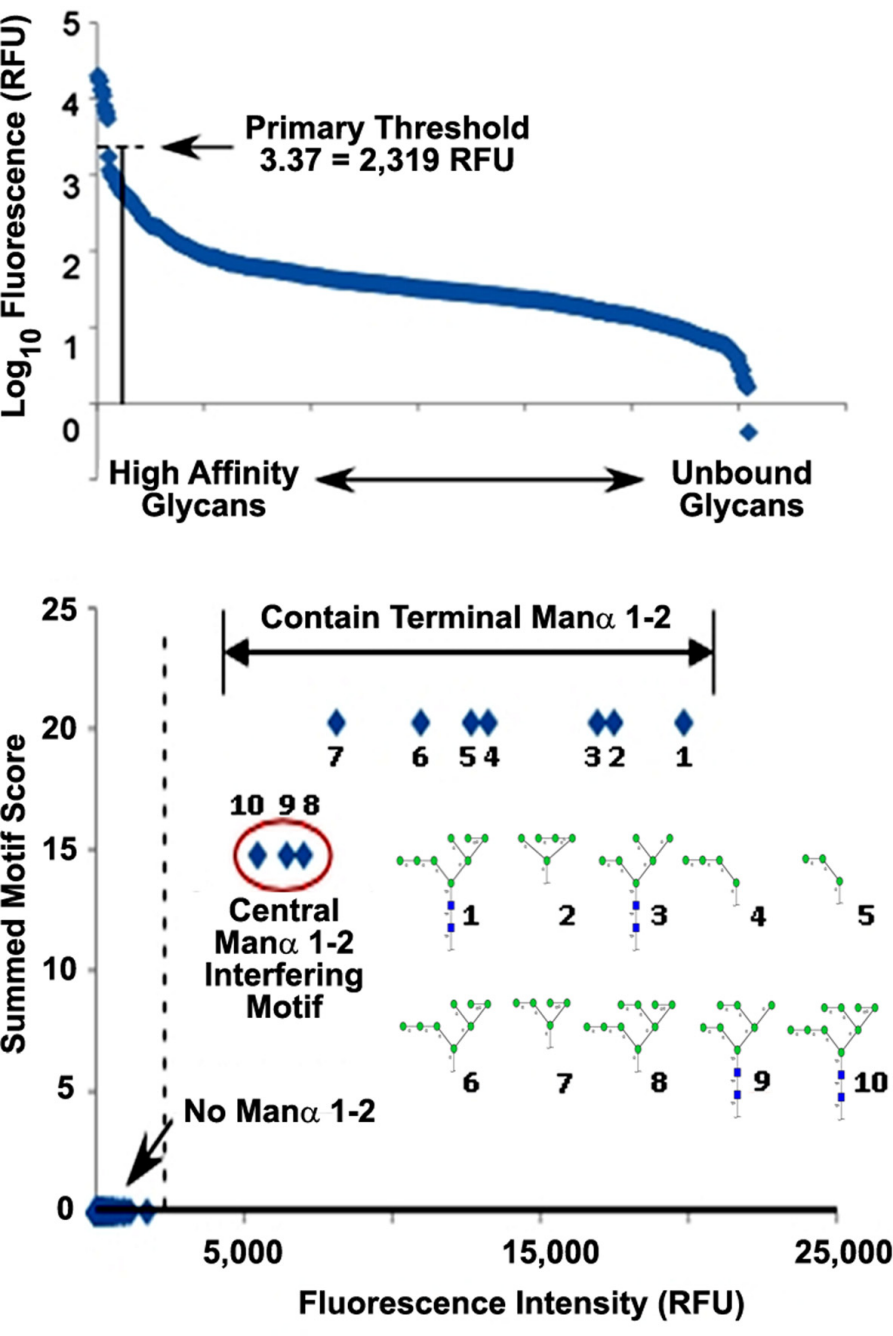
**Outlier Motif Analysis of the fine carbohydrate specificity of microvirin.** (Upper Panel) The fluorescence values for each glycan interaction exhibited by microvirin on the Consortium for Functional Glycomics Glycan Array #2340 at a concentration of 200 μg/mL were graphed in order of apparent intensity to determine the fluoresence threshold of positive interactions. Microvirin exhibited a clear demarcation between bound and unbound states on the array, and an unambiguous cut-off value of 2,319 relative fluorescence units (RFUs) was established. (Lower Panel) Outlier Motif Analysis was employed on the data set shown in (Upper Panel), according to the previously described method [40]. From this analysis it was determined that the presence of a Manα(1-2)-motif was necessary and sufficient for microvirin binding, while the presence of an intervening Manα1-2-linkage on the central arm of the chitobiose core structure of bound *N*-linked glycans reduced the affinity for microvirin for high-mannose structures. Using the refinement qualifier “Manα1,2 *NO* Manα1,2 on the central arm,” the summed motif scores for each glycan were plotted with respect to fluorescence intensity after detection with microvirin, which resulted in further segregation of high affinity carbohydrate motifs from the weaker affinity motifs. The dashed line represents the threshold for defining outliers, based on the distributions from all the glycans.

MVN has previously been shown to contain a single carbohydrate recognition domain (CRD) that can bind Manα(1–2)Man- linkages, the terminal disaccharide units on the arms of high-mannose-type *N*-linked oligosaccharides [38,39]. To more precisely and comprehensively determine the ligand specificity of the CRD of MVN, biotinylated MVN (at concentrations of 2, 20, and 200 μg/mL) was subjected to high-throughput glycan array screening among 611 structurally-defined carbohydrates by Core H of the Consortium for Functional Glycomics (CFG) utilizing Version 5.0 of the printed glycan microarray (Figure 1). The highest affinity carbohydrates from the 200 μg/mL data set are rank-ordered in Table 1. Unambiguous positive binding of MVN occurred with ten glycans, each of which contained the Manα(1–2)Man- motif (Figures 1A and 2).

**Table 1:** **High-affinity ligands of microvirin from the CFG's glycoarray analysis**

**Glycan #**	**Composition**	**Fluorescence (%)** ^**a**^
212	Manα1-2Manα1-6(Manα1-3)Manα1-6(Manα1-2Manα1-2Manα1-3)Manβ1-4GlcNAcβ1-4GlcNAcβ-Sp12	100.0
215	Manα1-2Manα1-2Manα1-6(Manα1-3)Manα-Sp9	78.1
216	Manα1-6(Manα1-3)Manα1-6(Manα1-2Manα1-3)Manβ1-4GlcNAcβ1-4GlcNAcβ-Sp12	72.9
208	Manα1-2Manα1-2Manα1-3Manα-Sp9	63.8
210	Manα1-2Manα1-3Manα-Sp9	57.7
316	Manα1-2Manα1-6(Manα1-3)Manα1-6(Manα1-2Manα1-2Manα1-3)Manα-Sp9	48.8
209	Manα1-2Manα1-6(Manα1-2Manα1-3)Manα-Sp9	39.5
317	Manα1-2Manα1-6(Manα1-2Manα1-3)Manα1-6(Manα1-2Manα1-2Manα1-3)Manα-Sp9	34.9
211	Manα1-6(Manα1-2Manα1-3)Manα1-6(Manα1-2Manα1-3)Manβ1-4GlcNAcβ1-4GlcNAcβ-Sp12	31.7
213	Manα1-2Manα1-6(Manα1-2Manα1-3)Manα1-6(Manα1-2Manα1-2Manα1-3)Manβ1-4GlcNAcβ1-4GlcNAcβ-Sp12	19.8

^a^Expressed as a percentage of the highest-affinity ligand, which exhibited a fluorescence reading of 19,838 RFU at 200 μg/mL microvirin.

To discriminate between primary and secondary binding specificities, we applied Outlier Motif Analysis to the 200 μg/mL MVN CFG data set. This analytical tool has been described previously as a method for refining the inherent specification of binding determinants from complex data sets such as those compiled from CFG glycan arrays [40]. From this analysis, MVN exhibited a clear demarcation between bound and unbound states (fluorescence threshold =2,319 RFU), with the Manα(1–2)Man- moiety serving as the common motif in all of the bound glycans (Figure 2A and B). Moreover, this motif was not present within any of the unbound constituents on the CFG glycan array. Other 2’ substituted mannose motifs, such as GlcNAcβ(1–2)Man-, a glycan well-represented within Version 5.0 of the CFG glycan array and a binding determinant for the *Lens culinaris* agglutinin (LCA) lectin, were not bound by MVN. Since the Manα(1–2)Man- motif adequately discriminated the bound versus unbound carbohydrates within the CFG data set for MVN, we then screened for motifs among the ten MVN-bound structures which might explain the varied binding within this group. Among the MVN-bound structures that contained a chitobiose core motif (GlcNAcβ [1-4] GlcNAc, i.e. *bona fide N*-glycans), the presence of an intervening Manα(1–2)Man linkage on the central arm of the *N*-glycan structure resulted in decreased MVN binding affinity (Figure 1B and Table 1). Strikingly, the two carbohydrate structures to which MVN bound with the highest- and lowest-affinity among the ten positive hits on the CFG glycan array varied by the absence or presence, respectively, of a single Manα(1–2)Man- substituent at this position (Figure 1B and Table 1). This observation may indicate that MVN exhibits preferential affinity for structural or rotational isomers with less restricted access to the Manα(1–2)Man- binding determinant (Figure 2B). This analysis validates the utility of MVN as a unique carbohydrate binding protein that specifically targets a structural motif that is present within the type of high-mannose *N*-glycans that are most commonly encountered in biological samples.

### Characterization of human GGT1 expressed in *Pichia pastoris* as a potential substrate pool that is exclusively modified with high-mannose-type *N*-glycans

**Figure 3:**
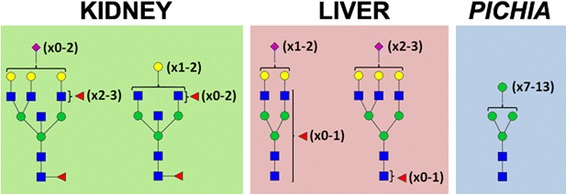
**Source-specific composition of** ***N*** **-glycans on human GGT1 (hGGT1).** A graphical summary of the predominant structural features of the *N*-glycans identified on hGGT1 expressed in normal human kidney (*left panel*) and liver (*middle panel*) tissues and *Pichia pastoris* (*right panel*) is shown. Kidney hGGT1 *N*-Glycans are dominated by complex type bisected bi- and triantennary structures with a high degree of fucosylation on core and peripheral GlcNAc residues and a low degree of sialylation. Liver hGGT1 *N*-glycans are dominated by complex-type biantennary structures with a high degree of sialylation and a low degree of core and peripheral fucosylation with modest contributions of triantennary structures. *Pichia pastoris*-expressed hGGT1 is modified by high-mannose-type *N*-glycans, ranging in size from Man_7_GlcNAc_2_ to Man_13_GlcNAc_2_. Symbols: *blue box*, GlcNAc; *green circle*, mannose; *yellow circle*, galactose; *red triangle*, fucose; *purple diamond*, *N*-acetylneuraminic acid.

**Figure 4:**
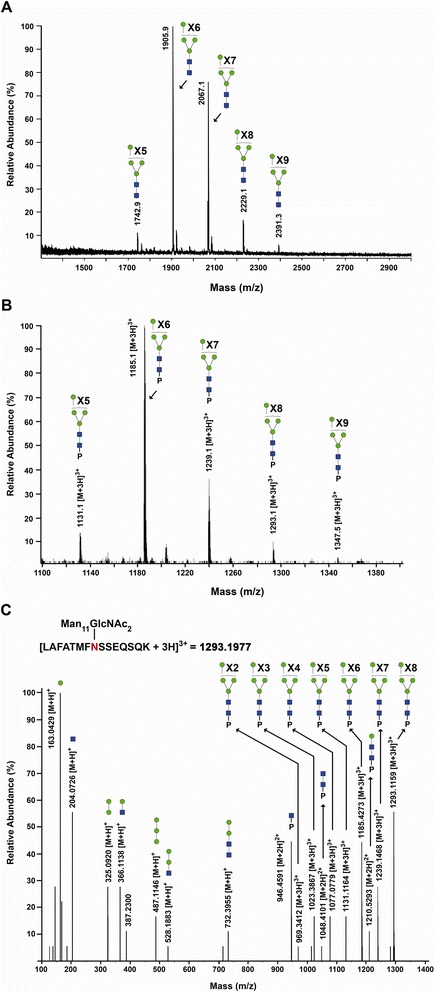
**Glycoconjugate analysis of hGGT1 expressed in** ***Pichia pastoris*** **. (A)** MALDI-TOF-MS profile of neutral *N*-glycans released by PNGase F from purified *Pp*-hGGT1. Ions corresponding to monosodiated *N*-glycans known to be expressed by *Pichia pastoris* are assigned. Symbols: *blue box*, GlcNAc; *green circle*, mannose. **(B)** Averaged LC/Q-Star MS spectrum for the Asn-120 (LAFATMFNSSEQSQK) family of glycopeptides from *Pp*-hGGT1, showing the identified glycoconjugates within their elution interval. *Asterisks* denote glycoconjugates that were confirmed by tandem MS analysis. P represents the LAFATMFNSSEQSQK tryptic peptide to which the *N*-glycans are attached. Unannotated peaks represent co-eluting nonglycosylated hGGT1 peptides. **(C)** MS/MS spectrum showing fragmentation of the Man_11_GlcNAc_2_-modified glycopeptide (*m*/*z* =1293.2) shown in **(B)**.

We previously showed that pools of hGGT1 purified from normal human kidney and liver tissue are each modified by a specific array of virtually non-overlapping complex-type *N*-glycans, with no high-mannose-type structures observed on hGGT1 glycopeptides originating from either of these tissues (Figure 3 and [17]). Indeed, high-mannose-type *N*-glycans on hGGT1 have only been observed in samples of diseased tissues from these organs [19,21]. To evaluate the utility of our hGGT1 microanalytical system and the diagnostic efficacy of our biotinylated MVN-reagent in assessing disease status, we generated hGGT1 modified with high-mannose-type structures. Others have shown that *Pichia pastoris N*-glycosylates recombinant human proteins with high-mannose-type structures [41-43], and we previously demonstrated that hGGT1 expressed in and purified from the yeast *Pichia pastoris* (*Pp*-hGGT1) is *N*-glycosylated and retains full enzymatic activity [15,16,44]. To investigate the *N*-glycan content of purified *Pp*-hGGT1, total *N*-glycans were enzymatically-released with the endoglycosidase PNGase F and profiled by MALDI-TOF/MS (Figure 4A). A family of ions were observed corresponding to predicted *N*-glycans that included Man_8_GlcNAc_2_ (4%), Man_9_GlcNAc_2_ (49%), Man_10_GlcNAc_2_ (37%), Man_11_GlcNAc_2_ (8%), and Man_12_GlcNAc_2_ (2%), consistent with findings from other recombinant human glycoproteins expressed in *Pichia pastoris* [41,43].

The distribution of *N*-glycans at different sites was addressed by LC-MS/MS profiling of tryptic glycopeptides, using diagnostic oxonium ions at *m*/*z* 163.0 and 204.1 to initially identify glycopeptide families, which elute as discrete chromatographic units [17]. As an example (Figure 4B), the *N*-glycans on the LAFATMFNSSEQSQK (Asn-120) hGGT1 glycopeptide approximately correspond to the range of high-mannose-type compositions found in total *Pp*-hGGT1 (Figure 4A). Collision-induced dissociation MS/MS was carried out to confirm the structure models, as illustrated by fragmentation (Figure 4C) of the triply-charged glycopeptide bearing a Man_11_GlcNAc_2_ glycan (*m*/*z* = 1293.2) to yield a spectrum of triply-, doubly- and singly-charged ions, including the core GlcNAc-modified peptide (*m*/*z* = 946.5) (Figure 4C). Similar *N*-glycan microheterogeneity was observed at Asn344 and Asn511, of the large and small subunits, respectively (Table 2). These results support the use of *Pp*-hGGT1 as a pool of hGGT1 that is uniformly modified by high-mannose type *N*-glycans (Figure 3).

**Table 2:** ***N*** **-glycan microheterogeneity of human GGT1 expressed in** ***Pichia pastoris*** **as determined by LC-MS/MS (see Figure** 4 **)**

**hGGT1**				
***N*** **-glycosylation**		**Theoretical**	**Mass**	**MS/MS**
**Site**	***N*** **-glycan**	**Mass**	**Measured**	**Support**
		[M + 3H] + 3		
N120	Man8GlcNAc2	1131.1307	1131.1153	✓
	Man9GlcNAc2	1185.1483	1185.1520	✓
	Man10GlcNAc2	1239.1659	1239.1576	✓
	Man11GlcNAc2	1293.1835	1293.1609	✓
	Man12GlcNAc2	1347.2011	1347.1885	✓
	Man13GlcNAc2	1401.2187	1401.2055	✓
		[M + 3H] + 3		
N344	Man7GlcNAc2	985.7410	985.7221	✓
	Man8GlcNAc2	1039.7586	1039.7490	✓
	Man9GlcNAc2	1093.7762	1093.7745	✓
	Man10GlcNAc2	1147.7938	1147.7869	✓
	Man11GlcNAc2	1201.8114	1201.8104	✓
	Man12GlcNAc2	1255.829	1255.8137	✓
		[M + 3H] + 3		
N511	Man8GlcNAc2	1112.8331	1112.8189	
	Man9GlcNAc2	1166.8507	1166.8450	✓
	Man10GlcNAc2	1220.8683	1220.8614	✓
	Man11GlcNAc2	1274.8859	1274.8865	✓
	Man12GlcNAc2	1328.9035	1328.8865	✓
	Man13GlcnNAc2	1382.9211	1832.9140	✓

**Figure 5:**
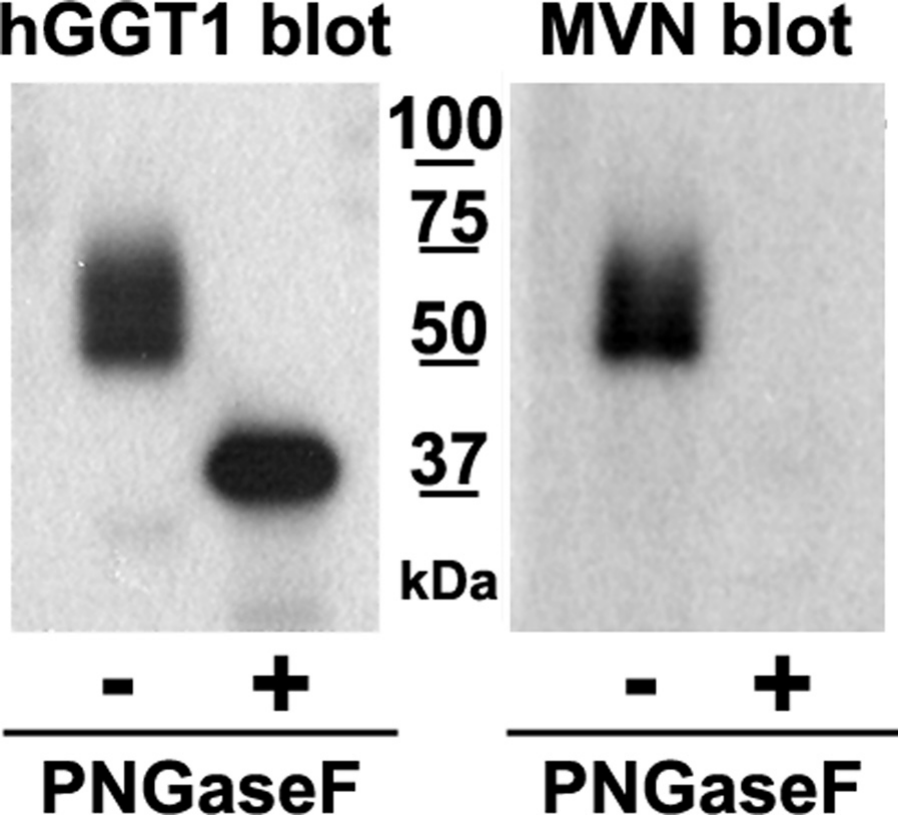
**MVN binds high-mannose type** ***N*** **-glycans on** ***Pp*** **-hGGT1.** Purified *Pp*-hGGT1 was incubated in the absence or presence of PNGaseF. *Pp*-hGGT1 (0.1 μg) from each reaction was resolved by SDS-PAGE, electroblotted onto nitrocellulose, and probed with either an antibody against the large subunit of hGGT1 (left panel) or with biotinylated-MVN (right panel). *MW*, molecular weight markers.

To evaluate whether purified *Pp*-hGGT1 is a high-affinity ligand of MVN, this pool was subjected to lectin and Western blot analyses using biotinylated MVN and an antibody specific for a peptide on the C-terminus of the large subunit of hGGT1. As shown in Figure 5, MVN and anti-hGGT1 similarly recognized the large subunit, which migrates broadly owing to microheterogeneity at the six *N*-glycosylation sites (Figure 5, *lanes 1 and 3* and [15]). Enzymatic deglycosylation collapses *Pp*-hGGT1 into a sharper band, which is no longer recognized by MVN (Figure 5, *lanes 2 and 4*). These results demonstrate that MVN recognizes *Pp*-hGGT1 by virtue of its *N*-glycans.

### Differential profiling of hGGT1 glycoepitopes by ALSA

**Figure 6:**
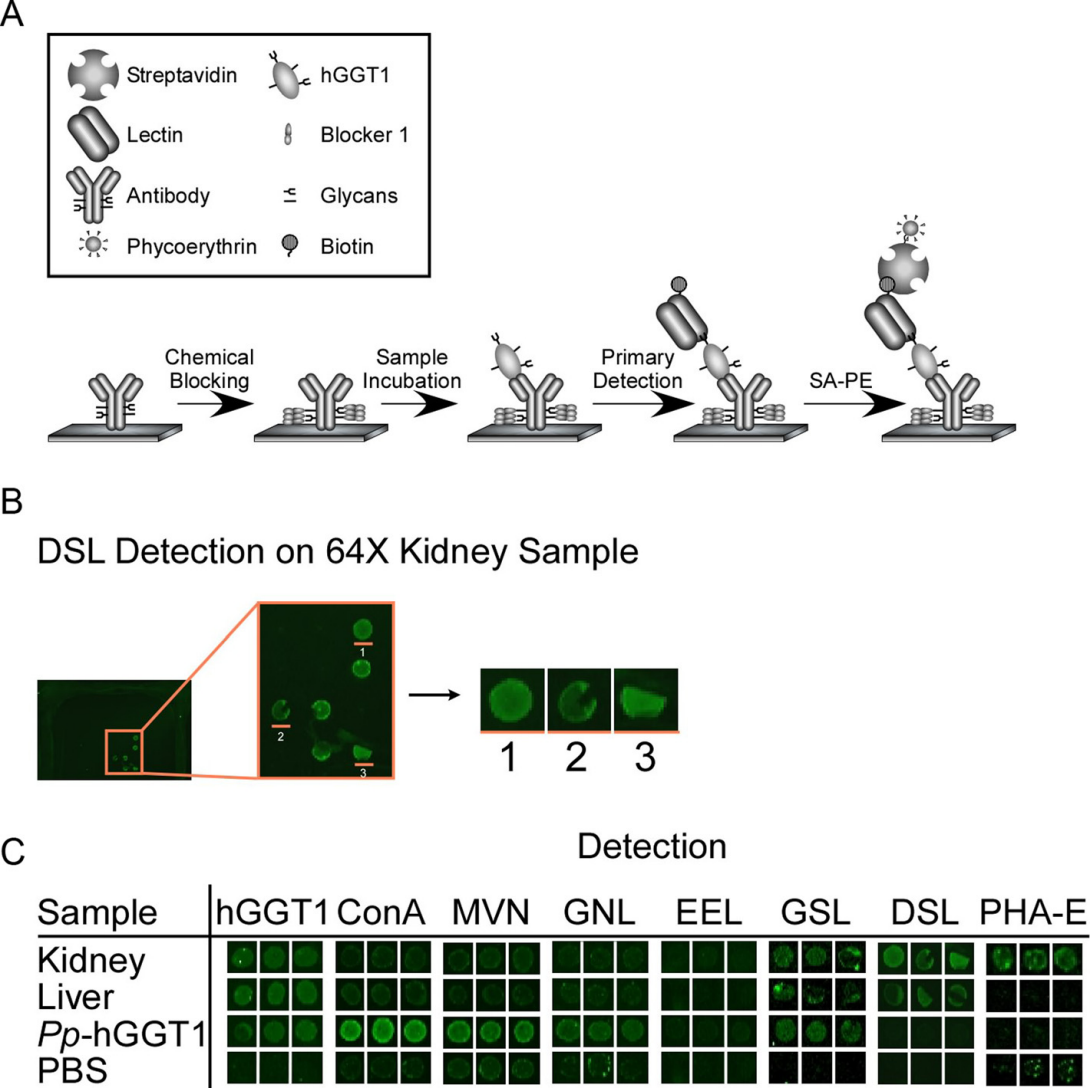
**Antibody lectin sandwich assay (ALSA). (A)** Graphical depiction of ALSA strategy. The hGGT1 antibodies are printed on a nitrocellulose slide and chemically derivatized to inactivate their glycans. hGGT1 is captured by the immobilized antibody and *N*-glycan features are probed using biotinylated lectins, which are subsequently detected using streptavidin-phycoerythrin and fluorescence scanning. **(B)** One printed antibody array well is shown with the magnified capture antibodies. Individual hGGT1 capture spots have been cut out to show triplicate detection of *Datura stramonium* lectin (DSL) among 64-fold kidney sample dilutions (equivalent to ~75 ng of total extracted kidney protein). **(C)** Representative results for hGGT1 capture antibody and detection reagents (indicated in the column labels).

We next investigated whether MVN and other lectins could be utilized in conjunction with ALSA technology to illuminate differences in carbohydrate content on the low quantities of hGGT1 present within complex biological samples. As depicted in Figure 6A, a previously-characterized polyclonal antibody (GGT129) that binds a C-terminal epitope in the large subunit of hGGT1 was immobilized and chemically-derivatized to prevent recognition by lectins or glycan-specific antibodies. The region containing the antibody epitope is not predicted to undergo post-translational modification [2].

**Figure 7:**
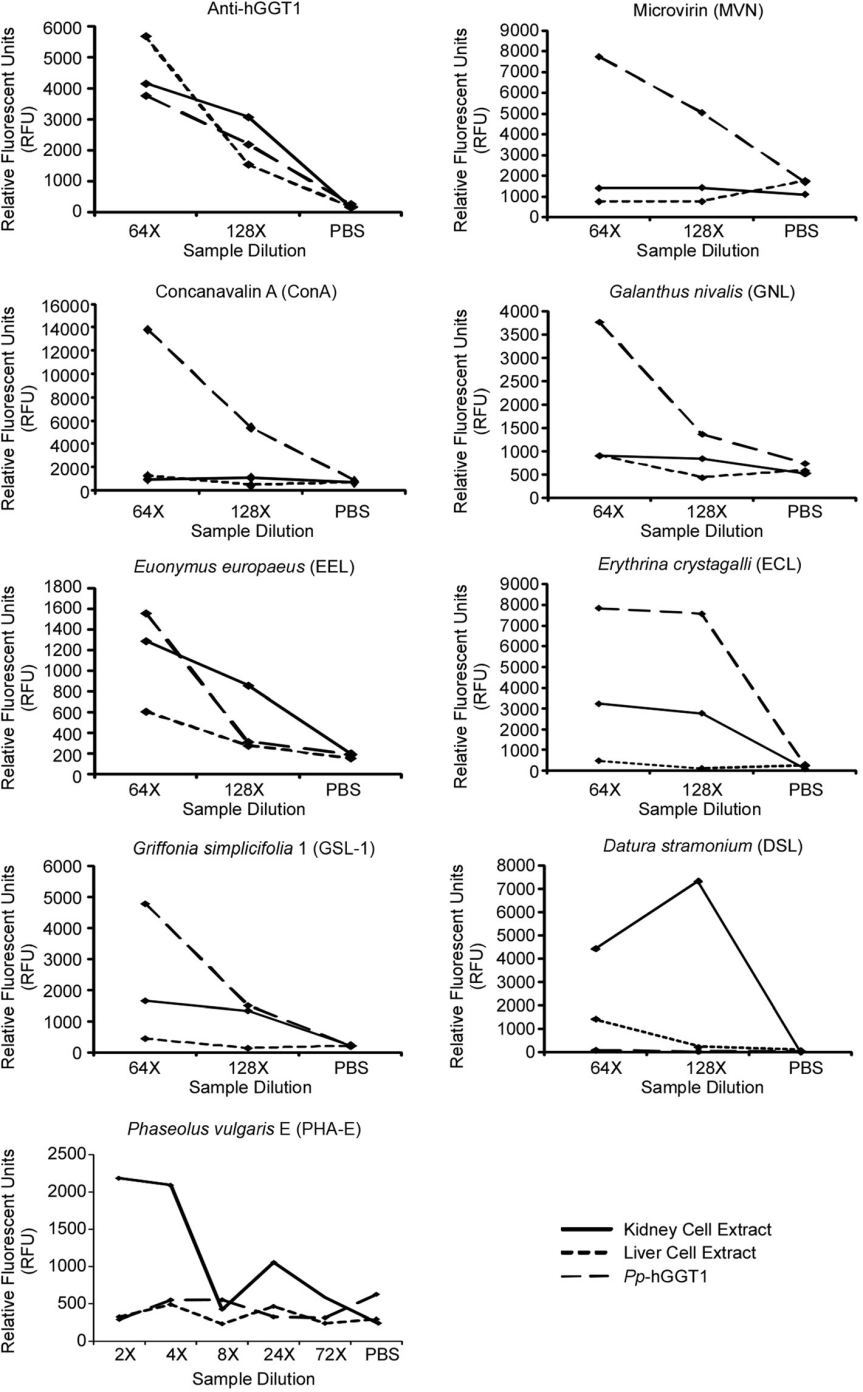
**Graphs of** ***Pp*** **-hGGT1, kidney hGGT1, and liver hGGT1 dilution series.** Each graph shows representative plots of dilutions of *Pichia pastoris*-expressed hGGT1, kidney extract hGGT1, and liver extract hGGT1 samples probed with the indicated lectins.

We first asked whether the immobilized hGGT1 antibody was able to capture the hGGT1 that was produced by normal human kidney tissue, normal human liver tissue, and *Pichia pastoris*. As shown in Figure 6C, the immobilized antibody immuno-captured equivalent amounts of hGGT1 from each pool of activity-normalized hGGT1, indicating that the method was appropriate for comparative analyses. Furthermore, a two-fold dilution of hGGT1 typically resulted in reduced signal (Figure 7), suggesting that lectin binding was approximately proportional to hGGT1 levels. From this analysis, a detection limit of approximately 0.3 nanograms for *Pichia pastoris*-expressed hGGT1 was achieved. Based on activity normalization of the sample pools and their similar dilution profiles, this also provides an estimate for the detection limits achieved for the immuno-captured hGGT1 in the more complex kidney and liver extracts, as well (Figure 7).

The results from probing the captured hGGT1s with a variety of glycan binding proteins are depicted in Figure 6 (panels B and C) and summarized in Table 3. MVN bound only *Pp*-hGGT1, consistent with exclusive expression of high-mannose type *N*-glycans on hGGT1 from this source. Concanavalin A (ConA), which exhibits strong affinity for high-mannose type *N*-glycans, also bound only *Pp*-hGGT1 (Figure 7). ConA also has weak affinity for complex type *N*-glycans, which we would expect to be present on the liver- and kidney-derived hGGT1, but under the conditions of our assay, no binding was observed on hGGT1 in these sample pools. *Galanthus nivalis* lectin (GNL), which like ConA also has strong affinity for high-mannose type *N*-glycans and weak affinity for complex *N*-glycans [45,46], also bound only *Pp*-hGGT1 (Figure 7). The similar binding patterns observed for MVN, ConA, and GNL are consistent with the presence of high-mannose type *N*-glycans on *Pp*-hGGT1 but not on the kidney- or liver-derived hGGT1.

**Table 3:** **Specificity of glycan binding proteins for hGGT1 from kidney, liver and** ***Pp*** **-hGGT1**

		**Signal 2X higher than background**		
Detection	Specificity	Kidney	Liver	*Pp*-hGGT1
Anti-GGT1	hGGT1	Yes	Yes	Yes
Microvirin (MVN)	Mannose	No	No	Yes
*Concanavalin A* (ConA)	terminal α-Man	No	No	Yes
*Galanthus nivalis* (GNL)	Man(terminal Manα(1-3)Man)	No	No	Yes
*Euonymus europaeus* (EEL)	Galα(1-3)Gal	Yes	No	Yes
*Erythrina cristagalli* (ECL)	Terminal Lac/LacNAc	Yes	No	Yes
*Griffonia simplicifolia* (GSL-1)	α-GalNAc, GalNAc α-Thr/Ser(T) and α-Gal	Yes	No	Yes
*Datura stramonium* (DSL)	(GlcNAc)n, polyLacNAc and LacNAc	Yes	Yes	No
*Phaseolus vulgaris* Erythroagglutinin(Pha-E)	Galb(1-4)GlcNAcβ(1-2)Man	Yes	No	No

*Euonymus europaeus* lectin (EEL) binds α-linked galactose primarily in the context of blood group B [Galα1-3(Fucα1-2)GlcNAc], and more weakly to α1-2 linked fucose [47,48]. As shown in Figure 7, EEL exhibited clear, concentration-dependent affinity for kidney-derived hGGT1. EEL also exhibited greater affinity for *Pp*-hGGT1 than liver-derived hGGT1 with increasing sample input (Figure 7). *Griffonia simplicifolia* Lectin 1(GSL-1) similarly binds terminal α-linked galactose but also α-linked GalNAc [49], and its affinity pattern for kidney- and liver-derived hGGT1 was similar to that measured for EEL at the higher sample dilution (Figure 7). However, unlike EEL, its affinity pattern for *Pp*-hGGT1 exceeded that observed for kidney-derived hGGT1 with increasing sample concentrations. ECL, which has specificity primarily for terminal Galβ1-4GlcNAc [50], exhibited high affinity for both *Pp*-hGGT1 and kidney-derived hGGT1 at both sample dilutions, although its apparent affinity for *Pp*-hGGT1 exceeded that for kidney hGGT1 on the array. Together, these results suggest the presence of terminal galactose, either α- or β-linked, on hGGT1 from kidney and *Pichia pastoris*. However, while we have demonstrated these glycan motifs on human kidney-derived hGGT1 (Figure 3 and [17]), we have no evidence that they are present on *Pp*-hGGT1, which is restricted to the production of high-mannose type carbohydrate motifs. Therefore, the affinity pattern of this particular subgroup of lectins for *Pp*-hGGT1 may arise from an inherent, yet cryptic, binding of high-mannose type *N*-glycans, a possibility that has recently been confirmed for EEL [51].

In marked contrast, *Datura stramonium* lectin (DSL) bound at saturating levels to kidney-derived hGGT1, weakly to liver-expressed hGGT1, and not at all to *Pp*-hGGT1 (Figures 6 and 7). The “hook effect” observed for DSL binding to kidney hGGT1, is commonly observed in immunoassays and other multivalent binding assays at saturating levels of the analyte [52]. DSL has specificity for *N*-acetyl-lactosamine (LAcNAc, Galβ1-4GlcNAc), as typically found on extended *N*-glycans [53]. This binding pattern is consistent with both the greater size and heterogeneity of complex type *N*-glycans which we identified on kidney-derived hGGT1 and demonstrated were not present on liver-derived hGGT1 or *Pp*-hGGT1 (Figures 3 and 4 and [17]).

*Phaseolus vulgaris*-Erythroagglutinin (Pha-E) specifically binds bisected *N*-linked glycans [54]. As Pha-E exhibited weaker binding affinity overall, we used higher concentrations (i.e. reduced dilutions) of hGGT1 for these analyses (Figures 6C and 7). The detection of bisecting GlcNAc on kidney-derived hGGT1 is consistent with our previous mass spectrometric analysis of hGGT1 isolated from this tissue source (Figure 3 and [17]). This type of modification is restricted to complex type *N*-glycans. Thus, the lack of interaction between Pha-E and *Pp*-hGGT1 is consistent with the expected absence of complex type *N*-glycans (Figure 4). Moreover, our mass spectrometric analysis did not detect bisected *N*-glycans on hGGT1 expressed in normal human liver, lending further credibility to the specificity of the ALSA platform and the specificity of the lectin binding pattern (Figure 3 and [17]).

### Lectin affinity blot validation of key source-specific differences in hGGT1 *N*-glycan content identified by ALSA microanalyses

ALSA analysis of hGGT1 expressed in *Pichia pastoris* and normal human kidney and liver revealed discriminatory lectin binding patterns that were consistent with documented differences in *N*-glycan content (Figure 3 and Table 3). To support these microanalytical findings, we investigated whether a subset of these binding patterns could be confirmed using the more time- and sample-exhaustive method associated with coupled immunoprecipitation and lectin blot analysis. Thus, hGGT1 was immunoprecipitated from activity-normalized aliquots of each class of hGGT1, and the bound fractions were subjected to SDS-PAGE and subsequent lectin blotting analysis with MVN, Pha-E, or DSL. ALSA analyses predicted that MVN would uniquely bind to *Pichia pastoris*-expressed hGGT1, while Pha-E would uniquely bind to kidney hGGT1 (Table 3). In the absence of a lectin that only bound liver hGGT1 in the ALSA platform, we used DSL, which exhibited high affinity for kidney-expressed hGGT1, modest affinity for liver-expressed hGGT1, and no apparent affinity for *Pichia pastoris*-expressed hGGT1 (Table 3).

**Figure 8:**
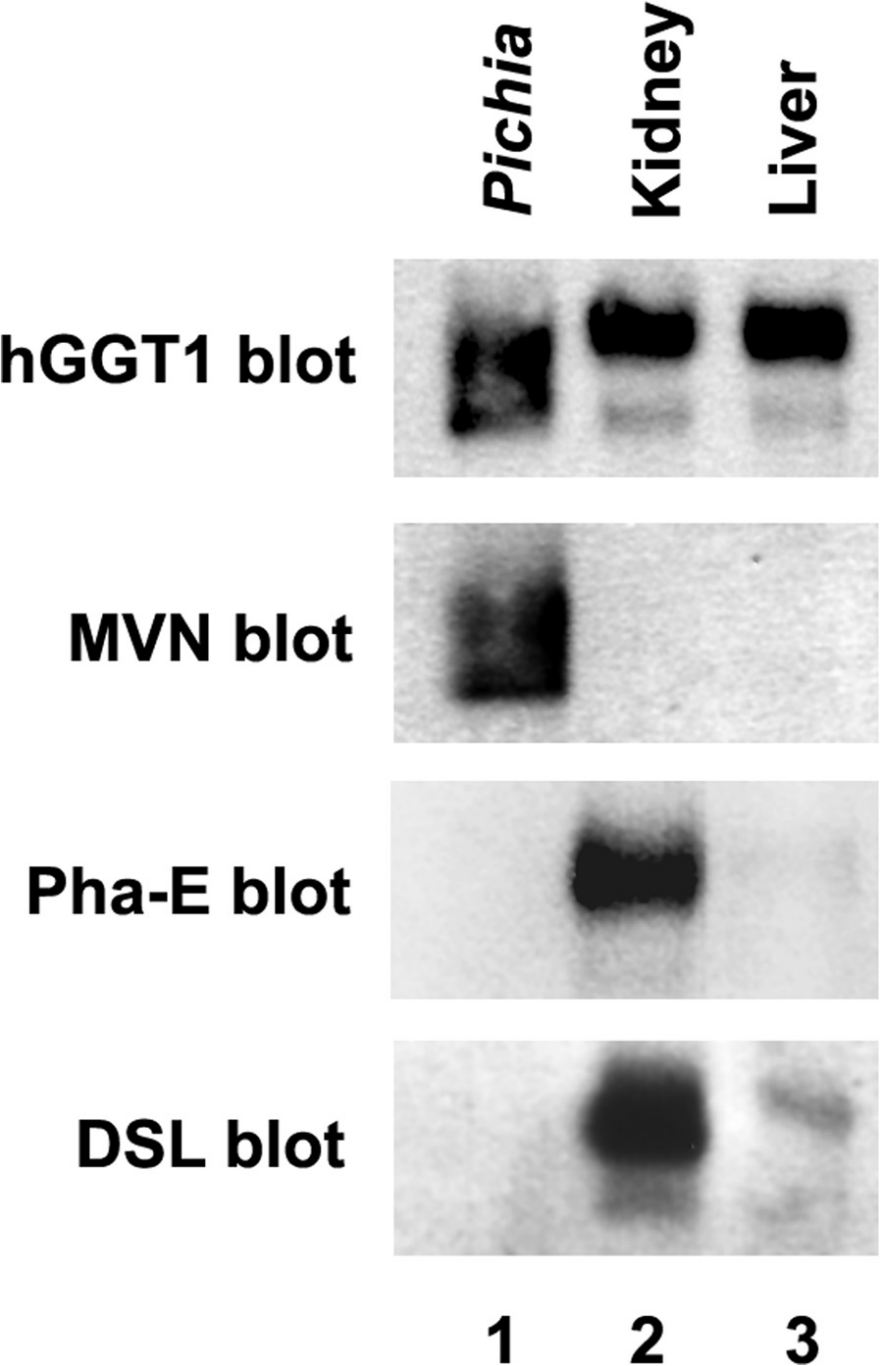
**hGGT1 lectin blotting confirms differential ALSA binding affinities.** Membrane extracts from normal human kidney and liver tissue or *Pichia pastoris*-expressed hGGT1 were activity-normalized and subjected to immunoprecipitation with a polyclonal anti-hGGT1 large subunit antibody. Equal volumes from each immunoprecipitation eluate were resolved by SDS-PAGE and affinity blotted with anti-hGGT1 (*hGGT1*, *top panel*) or the biotinylated lectins, microvirin (*MVN*, *second panel*), *Phaseolus vulgaris* Erythroagglutinin (*Pha*-*E*, *third panel*), and *Datura stramonium* lectin (*DSL*, *bottom panel*). Expanded views of the immunoblots shown here along with the relevant molecular weight markers are included in Additional file 1.

As shown in Figure 8, western blot analysis against hGGT1 confirmed that equivalent amounts of the protein were immuno-captured from the three samples and revealed that *Pp*-hGGT1 exhibits greater microheterogeneity in its migration pattern relative to kidney- or liver-derived hGGT1 (Figure 8, *top panel*). Previous studies from our lab showed that all six of the *N*-glycosylation sites on the large subunit of hGGT1 are occupied on the kidney- and liver-expressed enzyme, while only five are fully occupied on *Pp*-hGGT1 [15-17]. Therefore, these distinct banding patterns may reflect differences in glycosylation site occupancy or *N*-glycan composition. The apparent non-specific bands in the hGGT1 blot are likely the dissociated heavy chain of the rabbit IgG used to immunoprecipitate hGGT1 being recognized by the HRP-conjugated anti-rabbit secondary antibody used to visualize the hGGT1. In the ALSA analyses, the antibody that is printed is chemically-modified to prevent interactions between it and lectin probes or secondary antibodies [26,55]. MVN probing confirmed the ALSA data, showing specific recognition of *Pp*-hGGT1 (Figure 8, *MVN panel*, *lane 1*). Conversely, Pha-E exhibited strong, specific binding to kidney hGGT1 (Figure 8, *Pha*-*E panel*, *lane 2*), with no detectable binding to either *Pichia pastoris*- or liver-derived hGGT1 (Figure 8, *Pha*-*E panel*, *lanes 1 and 3*), again confirming the ALSA data. Lastly, DSL exhibited a strong binding preference for kidney hGGT1 and a modest interaction with liver hGGT1 (Figure 8, *DSL panel*, *lanes 2 and 3*), yet this lectin exhibited no apparent affinity for *Pichia pastoris*-expressed hGGT1 (Figure 8, *DSL panel*, *lane 1*). These results qualitatively recapitulate the quantitative evaluations from the ALSA microanalyses and support the application of ALSA as a valid approach for identifying differential hGGT1-specific lectin binding patterns in small aliquots of simple (e.g. purified *Pp*-hGGT1) and complex (e.g. tissue extracts) biological samples.

## Discussion

With the goal of miniaturizing comparative glycoanalyses on hGGT1, we incorporated the use of the highly-versatile ALSA platform to discern whether it was capable of reproducibly identifying differential lectin binding patterns among human kidney and liver tissue sample preparations of hGGT1, which have distinct *N*-glycan content. To augment the differential analysis, we generated a pool of hGGT1 in the yeast *Pichia pastoris* that we confirmed by mass spectrometry to be uniquely modified by high-mannose type *N*-glycans (Figures 3 and 4), a structural class of carbohydrates that are not observed on hGGT1 expressed in normal human kidney and liver tissues but have been documented on hGGT1 expressed in human malignancies [19,21]. We developed a biotinylated version of the *Microcystis aeruginosa* lectin MVN as a reagent that specifically interacts with high-mannose type *N*-glycans. With the aid of Core H of the Consortium for Functional Glycomics, we confirmed that MVN exhibits exquisite specificity for Manα(1–2)Man disaccharide, confirming and extending the original analyses conducted by Shahzad-ul-Hussan and colleagues on a more limited array of glycans [39]. Using outlier motif analysis [40], we gained new insights into the fine saccharide binding specificity of the carbohydrate recognition domain of MVN. Our analysis suggests that an intervening Manα(1–2)Man substituent comprising the central (B) arm of high-mannose structures significantly reduces the affinity of MVN (Figure 1B and Table 1). The affinity pattern of MVN is distinct from the high-mannose binding lectins GNL and ConA, which can cross-react with complex type *N*-glycans [45,46].

With the help of this new reagent, we demonstrated that the ALSA platform could reveal clear, precise distinctions in the glycan content on hGGT1 immuno-captured from three distinct sources. Despite significant differences in the complexity of the hGGT1 samples, the immobilized hGGT1 antibody reproducibly captured the target glycoprotein with equivalent efficiency, demonstrating that there was no inherent technical bias in this phase of the analysis (Figures 6C and 7). Moreover, this observation underscores the capacity of this platform to accommodate sample preparations with variable enrichment of the analytical target. In general, the lectin binding patterns illuminated by the ALSA analysis were consistent with known, quantifiable differences in the glycan content on hGGT1 that we had previously identified by mass spectrometry (Figure 4 and [17]). However, unlike other complementary approaches, discriminatory distinctions in glycan content could be detected on hGGT1 using sub-microgram quantities of total protein within the complex tissue sample pools, and in the case of the *Pp*-hGGT1, sub-nanogram amounts of total protein were successfully analyzed. As expected, lectins (MVN, GNL, and ConA) with high affinity for high-mannose structures exhibited strong, preferential binding for immuno-captured *Pp*-hGGT1, which is exclusively modified with high-mannose structures (Figures 4 and 7 and Table 3). In contrast, Pha-E exhibited unique affinity for kidney-expressed hGGT1 (Figure 7 and Table 3). Pha-E is selective for complex type *N*-glycans containing bisecting GlcNAc motifs [54]. This structural feature was predicted by previous mass spectrometric and enzymatic analyses to be unique to the kidney hGGT1 pool examined in this study (Figure 3 and [17,19]), providing validity to the differential ALSA binding pattern. DSL, on the other hand, exhibited high affinity for kidney hGGT1, moderate affinity for liver hGGT1, and no detectable affinity for *Pichia pastoris*-expressed hGGT1 by ALSA (Figure 7 and Table 3). In light of the fact that DSL is known to bind *N*-acetyl-lactosamine motifs (Galβ1-4GlcNAc) [56,57], a feature unique to hybrid or complex type *N*-glycans, the ALSA binding pattern exhibited by this lectin is consistent with the greater size and complexity of *N*-glycans on kidney hGGT1 relative to those identified on the liver enzyme and with the absence of these structural motifs on the *Pichia pastoris*-expressed enzyme (Figure 4 and [17]). These patterns were faithfully replicated in standard Western blots of immunoprecipitated hGGT1 samples, albeit consuming approximately 60 times more primary sample. Therefore, these results not only provide proof of concept support for the validity of the ALSA platform in detecting differences in glycosylation patterns on hGGT1 but also demonstrate that primary screening with ALSA using limited amounts of biological material can lead to responsible conservation of primary samples that can subsequently be used for other complementary targeted profiling techniques. A broader search of lectins, or application of lectin arrays [58], is needed to identify ALSA-exploitable lectins that detect features specific to liver-derived hGGT1. However, liver glycosylation frequently includes sialic acid [17,59], whose negative charges can inhibit the binding of certain lectins. The lectins in this study do not bind sialylated motifs. We probed for sialic acid modifications on the immunocaptured hGGT1, but we had only low binding with lectins that detect sialylation. We are working to improve the binding of these lectins in the ALSA. Quantitative binding data from an expanded lectin pool may predict glycan motifs that are unique to hGGT1 from each of these sources [60]. Interestingly, GSL-1 and ECL, which are known for Gal-associated motifs not expected on *Pichia pastoris*-expressed glycoproteins, exhibited substantial binding to *Pp*-hGGT1 (Figures 6C and 7). However, even these lectins show weak cross-reactivity with certain high mannose *N*-glycans in publically available databases (www.functionalglycomics.org). This suggests that certain *N*-glycans formed by the *Pichia pastoris* strain employed here are able to adopt a conformation that can be recognized by GSL-1 and ECL.

## Conclusion

Based on the promising attributes of this analytical approach, we anticipate that this adapted version of the ALSA platform can be applied to directly survey the manner and extent in which altered glycosylation patterns on hGGT1 positively correlate with various primary malignancies and other relevant pathologies in order to evaluate their potential for providing diagnostic or prognostic information that might assist with clinical intervention strategies.

## Methods

### Microvirin purification

The nucleotide sequence for the open-reading frame (ORF) of *MVN* (EMBL accession AM041066) was synthesized with flanking NdeI and BamHI restriction sites in the pUC57 shuttle vector GenScript (Piscataway, NJ). The *MVN* ORF was transferred into the *E. coli* expression construct pET-15b (Invitrogen, Grand Island, NY), using NdeI and BamHI, where it was expected to encode the full-length protein with an N-terminal hexahistadine tag. The resultant construct was transformed into *E. coli* BL21 (DE3) and transformants were isolated at 37°C on Luria-Bertani (LB) agarose media containing 100 μg/mL ampicillin. A clonal transformant was propagated at 37°C to an OD_600_ of ~0.6. The culture was then cooled to room temperature in a water bath, supplemented with isopropyl β-D-1-thiogalactopyranoside (IPTG) to 0.5 mM, and shaken for another 3 h at 25°C. Cells were harvested at 4,000 × *g* for 15 min at room temperature, resuspended in Binding Buffer (50 mM sodium phosphate, pH 8.0, 300 mM NaCl, 1 μM leupeptin, 1 μg/mL aprotinin) containing 10 mM imidazole, and lysed in an ice-cold French Press. The lysate was centrifuged at 10,000 × *g* for 20 min at 4°C, and the clarified supernatant loaded onto PerfectPro Ni-NTA (5Prime, Gaithersburg, MA) column. The column was washed with 20 bed volumes of Binding Buffer containing 20 mM imidazole, and the affinity-bound protein was eluted with Binding Buffer containing 250 mM imidazole. Fractions containing affinity-purified MVN [as monitored by sodium dodecyl sulfate-polyacrylamide gel electrophoresis (SDS-PAGE)] were pooled and dialyzed against phosphate-buffered saline (PBS: 10 mM Na_2_HPO_4_, 1.8 mM KH_2_PO_4_, 137 mM NaCl, 2.7 mM KCl, pH 7.4). MVN was biotinylated using the EZ-Link NHS-SS- Biotinylation kit (Pierce, Rockland, IL), according to the manufacturer’s protocol, and then fractionated away from unconjugated biotin on a Sephadex G-25 (Sigma-Aldrich, St. Louis, MO) column equilibrated in PBS. The resulting biotinylated MVN stock (1.0 mg/mL) was supplemented with 0.05% sodium azide and stored at 4°C.

### Glycan array analysis

The glycan array data were collected by Core H of the Consortium for Functional Glycomics (CFG), using biotinylated MVN and Alexa Fluor 488-conjugated *avidin* (Invitrogen) on Version 5.0 of the printed glycan array, according to protocols that were published previously [61]. Version 5.0 of the glycan array consists of 611 unique glycans in replicates of six.

### Purification of human GGT1 from *Pichia pastoris*

Recombinant glycosylated human GGT1 was affinity-purified from *Pichia pastoris* X-33 strain [16,44], and aliquots (0.25 mg/mL) were stored at −80°C in 25 mM Tris–HCl, 1 mM dithiothreitol, 0.5 mM EDTA, pH 8.0.

### Mass spectrometric analyses

#### Release of N-Glycans

*N*-glycans were released from 5 μg of recombinant hGGT1 using peptide:N-glycosidase F (PNGase F, New England Biolabs, Ipswich, MA) and then purified, using Sep-Pak C18 columns (Waters Corp., Milford, MA) and activated charcoal Carbograph cartridges (Harvard Apparatus, Holliston, MA). The *N*-glycan pool was then dried and resuspended in 20 μL of 50% methanol/50% H_2_O, as described previously [62].

#### Glycomic analysis

*N*-glycans (0.5 μL) were analyzed by MALDI-TOF-MS in positive ion mode on an Ultraflex II mass spectrometer (Bruker Daltonics, Billerica, MA) with a SMARTbeam laser, using 2,5-dihydroxybenzoic acid, as described [62]. A dextrin ladder (*M*_r_ 500–3000, V-laboratories, Covington, LA) was spotted and analyzed in parallel as an external mass calibrant. Mass accuracy was typically <50 ppm for glycans.

#### Glycopeptide analysis

Tryptic digests were prepared and analyzed on a QSTAR Elite hybrid quadrupole-TOF mass spectrometer (Applied Biosystems, Grand Island, NY) equipped with a nanospray ionization source (Ultimate 3000, Dionex, Sunnyvale, CA), as described previously [62]. Briefly, 1.2 μg of trypsinized protein was eluted from a C18 column using acetonitrile/TFA directly into the mass spectrometer programmed to perform MS/MS fragmentation on the three most abundant ions under data-dependent conditions. Candidate glycopeptides were manually identified within the data set by probing the entire LC/MS-MS run for mass matches against predicted glycopeptides and expanded by the inclusion of glycans of related compositions, as informed by the glycans identified in the glycomics data. Assuming equal ionization efficiency of each glycoform at a particular *N*-glycosylation site, the relative abundance of each glycoform on a single glycosylation site was determined by summing the total ion intensities for all of the glycoforms within a glycopeptide family over the entire elution window. Each total ion intensity was then calculated as a percentage relative to the other family members. The reported percentages represent the average of at least three separate LC/MS-MS experiments.

### Tissue extracts and sample preparations

Normal human kidney and liver tissues were obtained from the National Disease Research Interchange (Philadelphia, PA) and stored at −80°C. CHAPS-solubilized membrane fractions containing hGGT1 were prepared as described previously [17]. Using a standardized quantitative biochemical assay for GGT1 transpeptidation activity [44], tissue extracts and aliquots of *Pichia pastoris*-expressed hGGT1 were volume-normalized, such that equivalent total units of hGGT1 activity from each sample pool were diluted into 25 mM Tris–HCl buffer (pH 7.4) containing 0.5% CHAPS. The total protein concentrations of the activity-normalized samples used for ALSA analyses were 4.8, 25.5, and 0.007 mg/mL for kidney, liver, and *Pichia pastoris*-expressed hGGT1 preparations, respectively. These studies were approved by the Institutional Review Board at the University of Oklahoma Health Sciences Center under IRB Protocol #11000.

### Microarray fabrication and preparation

Antibody microarrays were prepared as previously described [55]. A microarrayer (2470 Arrayer, Aushon Biosystems, Billerica, MA) was used to spot subnanoliter droplets of each antibody solution, prepared at 250 μg/mL in 1X PBS, on the surfaces of ultrathin nitrocellulose-coated glass microscope slides (PATH slides, Grace Bio-Labs, Bend, OR). Forty-eight identical arrays were printed on each slide, with each array consisting of the antibody targeting hGGT1, as well as negative and positive control antibodies, printed in six replicates. A wax border was imprinted around each of the arrays to define hydrophobic boundaries (SlideImprinter, The Gel Company, San Francisco, CA). For chemical derivatization of the printed antibodies, each slide was incubated in 150 mM sodium metaperiodate diluted in 0.02 M sodium acetate (pH 5.5) for two hours at 4°C, washed in 0.02 M sodium acetate (pH 5.5) for three minutes at room temperature, and then incubated in 0.02 M sodium acetate containing 10 mM L-glutamic acid for two hours at room temperature. Slides were then washed three times in PBS containing 0.5% Tween-20, centrifuged briefly, and then were stored at 4°C in a desiccated, vacuum-sealed slide box until use.

### Sandwich assays

Assays were performed similar to previously described methods [26,55]. Aliquots of volume-and activity-normalized stocks of kidney and liver tissue extracts or *Pichia pastoris*-expressed hGGT1 were supplemented with SDS to a final concentration 0.5% and denatured at 100°C for 10 minutes in order to dissociate hGGT1 from co-purifying membrane-bound glycoproteins. Samples were then cooled to room temperature and then diluted two-fold into a PBS buffer containing 0.1% Brij, 0.1% Tween-20 and 50 μg/mL of protease inhibitor. An IgG/IgY cocktail consisting of a final concentration of 400 μg/mL goat and sheep IgG and 800 μg/mL rabbit and mouse IgG (Jackson Immunoresearch, West Grove, PA) was added to each sample to eliminate non-specific binding to the printed antibodies. Slides were blocked in a solution containing PBS-0.5% Tween-20 buffer with the addition of 1% BSA. Six microliters of sample were then applied to each array overnight at 4°C. The arrays were washed in three changes of PBS/0.1% Tween-20 for three minutes each and dried by centrifugation (Eppendorf 5810R, rotor A-4-62, 1500 × g for three minutes). Captured antigens were detected with biotinylated detection reagents at a concentration of 1–10 μg/mL for one hour at room temperature followed by incubation with 1 μg/mL streptavidin-phycoerythrin (Roche Applied Science, Indianapolis, IN). The slides were washed and dried as above and scanned for fluorescence emission at 575 nm using a microarray scanner (LS Reloaded, TECAN, Durham, NC). At least three independent sample array analyses were conducted for each ALSA experiment. The data analysis and preparation was performed using Microsoft Excel and Canvas X.

### Lectin blots

Equivalent units of hGGT1 activity from *Pichia pastoris*-expressed hGGT1 stocks and from normal human kidney and liver tissue extracts were subjected to immunoprecipitation with GGT129 antibody and bovine serum albumin-blocked protein G-Sepharose beads (GE Healthcare, Piscataway, NJ), as described [15]. Bound proteins were eluted in 50 μL of Laemmli sample buffer (2% SDS, 5% glycerol, 5% 2-mercaptoethanol, 0.002% bromphenol blue, 62.5 mM Tris–HCl, pH 6.8) for 10 min at 100°C, and five microliter aliquots of each eluate were resolved on 8% SDS-PAGE gels. The resolved proteins were electroblotted onto nitrocellulose membranes and blocked overnight at 4°C in Carbo-Free blocking agent (Vector Labs, Burlingame, CA). Lectin blotting was conducted using the appropriate biotinylated lectins diluted in PBS containing 1 mM calcium chloride and 0.05% Tween-20 (PBST). Each membrane was incubated in PBST with either 1.0 μg/mL biotinylated MVN (described herein), 1.0 μg/mL biotinylated *Datura stramonium* lectin (DSL, Vector Labs), or 0.2 μg/mL biotinylated *Phaseolus vulgaris*-Erythroagglutinin (Pha-E, Vector Labs) for 1 h at room temperature. Membranes were then washed six times for five minutes with PBST and incubated with 0.5 μg/mL horseradish peroxidase-conjugated avidin (avidin-HRP, Vector Labs) for 1 h at room temperature in PBST. The membranes were subjected to an additional round of washing with PBST, and the blots were visualized by chemiluminescence, according to the manufacturer's protocol (ECL Plus, GE Healthcare). Western blotting against the large subunit of GGT1 was carried out as described previously [15].

## Additional file

Additional file 1: Figure S1. Expanded view of Figure 8, hGGT1 lectin blotting confirms differential ALSA binding affinities. Membrane extracts from normal human kidney and liver tissue or *Pichia pastoris*-expressed hGGT1 were activity-normalized and subjected to immunoprecipitation with a polyclonal anti-hGGT1 large subunit antibody. Equal volumes from each immunoprecipitation eluate were resolved by SDS-PAGE and affinity blotted with anti-hGGT1 (*hGGT1 blot*) or the biotinylated lectins, microvirin (*MVN blot*), *Phaseolus vulgaris* Erythroagglutinin (*Pha*-*E blot*), and *Datura stramonium* lectin (*DSL blot*). Position of molecular weight markers are shown on right.

## Abbreviations

ALSA: Antibody-lectin sandwich arrays
CFG: Consortium for Functional Glycomics
ConA: Concanavalin A the *Canavalia ensiformis* lectin
CRD: Carbohydrate recognition domain
DSL: *Datura stramonium* lectin
EEL: *Euonymus europaeus* lectin
GGT1: γ-Glutamyl transpeptidase 1
GNL: *Galanthus nivalis* lectin
GSL1: *Griffonia simplicifolia* lectin 1
hGGT1: human GGT1
IPTG: Isopropyl β-D-1-thiogalactopyranoside
LCA: *Lens culinaris* agglutinin lectin
MVN: Microvirin
ORF: Open-reading frame
Pha-E: *Phaseolus vulgaris*-Erythroagglutinin
*Pp*-hGGT1: Human GGT1 expressed in *Pichia pastoris*
