# Supplementary material for: Detection of distinct glycosylation patterns on human γ-glutamyl transpeptidase 1 using antibody-lectin sandwich array (ALSA) technology
Source: BMC Biotechnol. 2014 Dec 6;14:101. doi: 10.1186/s12896-014-0101-0 (PMC4297448; doi:10.1186/s12896-014-0101-0)
Supplement: Additional file 1: Figure S1. — Expanded view of Figure 8, hGGT1 lectin blotting confirms differential ALSA binding affinities. Membrane extracts from normal human kidney and liver tissue or Pichia pastoris-expressed hGGT1 were activity-normalized and subjected to immunoprecipitation with a polyclonal anti-hGGT1 large subunit antibody. Equal volumes from each immunoprecipitation eluate were resolved by SDS-PAGE and affinity blotted with anti-hGGT1 (hGGT1 blot) or the biotinylated lectins, microvirin (MVN blot), Phaseolus vulgaris Erythroagglutinin (Pha-E blot), and Datura stramonium lectin (DSL blot). Position of molecular weight markers are shown on right. [file 12896_2014_101_MOESM1_ESM.docx]

Additional file 1: Figure S1. **Expanded view of Figure 8, hGGT1 lectin blotting confirms differential ALSA binding affinities.** Membrane extracts from normal human kidney and liver tissue or *Pichia pastoris*-expressed hGGT1 were activity-normalized and subjected to immunoprecipitation with a polyclonal anti-hGGT1 large subunit antibody. Equal volumes from each immunoprecipitation eluate were resolved by SDS-PAGE and affinity blotted with anti-hGGT1 (*hGGT1 blot)* or the biotinylated lectins, microvirin (*MVN blot*), *Phaseolus vulgaris* Erythroagglutinin (*Pha-E blot*), and *Datura stramonium* lectin (*DSL blot).* Position of molecular weight markers are shown on right.
